# Supplementary material for: Curated and harmonised transcriptomics datasets of interstitial lung diseases
Source: Data Brief. 2025 Oct 14;63:112139. doi: 10.1016/j.dib.2025.112139 (PMC12581653; doi:10.1016/j.dib.2025.112139)
Supplement: Supplementary file 1 [file mmc1.zip › Supplementary_material/RNA-seq/fastQC reports/GSE138283/SRR10212994_fastqc.html]

SRR10212994.fastq.gz FastQC Report 

FastQC Report

Fri 2 Feb 2024  
SRR10212994.fastq.gz

## Summary

- Basic Statistics
- Per base sequence quality
- Per sequence quality scores
- Per base sequence content
- Per sequence GC content
- Per base N content
- Sequence Length Distribution
- Sequence Duplication Levels
- Overrepresented sequences
- Adapter Content

## Basic Statistics

| Measure | Value |
| --- | --- |
| Filename | SRR10212994.fastq.gz |
| File type | Conventional base calls |
| Encoding | Sanger / Illumina 1.9 |
| Total Sequences | 58548928 |
| Sequences flagged as poor quality | 0 |
| Sequence length | 35-76 |
| %GC | 46 |

## Per base sequence quality

## Per sequence quality scores

## Per base sequence content

## Per sequence GC content

## Per base N content

## Sequence Length Distribution

## Sequence Duplication Levels

## Overrepresented sequences

| Sequence | Count | Percentage | Possible Source |
| --- | --- | --- | --- |
| CCTTAGGCAACCTGGTGGTCCCCCGCTCCCGGGAGGTCACCATATTGATG | 292769 | 0.5000416062271883 | No Hit |
| CCCCACTACCACAAATTATGCAGTCGAGTTTCCCACATTTGGGGAAATCGCAGGGGTCAGCACATCCGGAGTGCA | 251611 | 0.42974484520024003 | No Hit |
| GTCGAGTTTCCCACATTTGGGGAAATCGCAGGGGTCAGCACATCCGGAGTGCAATGGATAAGCCTCGCCCTGGGA | 202651 | 0.34612247725526246 | No Hit |
| CCACAAATTATGCAGTCGAGTTTCCCACATTTGGGGAAATCGCAGGGGTCAGCACATCCGGAGTGCAATGGATA | 198126 | 0.3383938985185177 | No Hit |
| CCCACTACCACAAATTATGCAGTCGAGTTTCCCACATTTGGGGAAATCGC | 191608 | 0.32726132919120227 | No Hit |
| CACAAATTATGCAGTCGAGTTTCCCACATTTGGGGAAATCGCAGGGGTCA | 172518 | 0.29465612077474757 | No Hit |
| CACTACCACAAATTATGCAGTCGAGTTTCCCACATTTGGGGAAATCGCAG | 138203 | 0.23604702036559919 | No Hit |
| CTCAGACCGCGTTCTCTCCCTCTCACTCCCCAATACGGAGAGAAGAACGATCATCAATGGCTGACGGCAGTTGC | 135942 | 0.23218529295702903 | No Hit |
| GCTATTCACAGGCGCGATCCCACTACTGATCAGCACGGGAGTTTTGACCT | 112293 | 0.1917934347149789 | No Hit |
| GGCTGGAGTGCAGTGGCTATTCACAGGCGCGATCCCACTACTGATCAGCA | 106776 | 0.1823705465623555 | No Hit |
| GCTCCGTTTCCGACCTGGGCCGGTTCACCCCTCCTTAGGCAACCTGGTGGTCCCCCGCTCCCGGGAGGTCACCAT | 105284 | 0.1798222505457316 | No Hit |
| GTCGATGCGTGGAGTGGACGGAGCAAGCTCCTATTCCATCTCCTATTTCC | 105158 | 0.17960704592234378 | No Hit |
| GTTTCCGACCTGGGCCGGTTCACCCCTCCTTAGGCAACCTGGTGGTCCCC | 103274 | 0.1763892244107356 | No Hit |
| GTGCGATCGGCCCGAGGTTATCTAGAGTCACCAAAGCCGCCGGCGCCCGC | 102114 | 0.17440797549700654 | No Hit |
| GTCCCCCACTACCACAAATTATGCAGTCGAGTTTCCCACATTTGGGGAAA | 99093 | 0.16924818845530357 | No Hit |
| CTTAGGCAACCTGGTGGTCCCCCGCTCCCGGGAGGTCACCATATTGATGC | 90165 | 0.1539994037123959 | No Hit |
| GTCGATGCGTGGAGTGGACGGAGCAAGCTCCTATTCCATCTCCCTGCTCC | 89874 | 0.15350238351076215 | No Hit |
| CCACTACCACAAATTATGCAGTCGAGTTTCCCACATTTGGGGAAATCGCA | 83360 | 0.14237664607625267 | No Hit |
| GCTCAGGCTGGAGTGCAGTGGCTATTCACAGGCGCGATCCCACTACTGAT | 78634 | 0.1343047647260083 | No Hit |
| GGCTATTCACAGGCGCGATCCCACTACTGATCAGCACGGGAGTTTTGACC | 72441 | 0.12372728668917729 | No Hit |
| GTGGCTATTCACAGGCGCGATCCCACTACTGATCAGCACGGGAGTTTTGA | 62292 | 0.10639306666724965 | No Hit |
| GTCTGGAGTCTTGGAAGCTTGACTACCCTACGTTCTCCTACAAATGGACC | 59068 | 0.1008865610656441 | No Hit |

## Adapter Content

Produced by FastQC (version 0.11.7)
